# Mourning

**Published:** 1890-03-22

**Authors:** 


### Words of Consolation.

### MOURNING.

God's Holy Word assures us that mourners shall be comforted, and a special promise is given by our Lord Himself to the sorrowing, whose state He describes as one of blessedness. Sad and dreary their lot may appear in the first agony of despair, and the prayed-for relief may tarry, yet their sorrow will yield. For as one whom his mother comforteth so will He, the Man of Sorrows, comfort; their bleeding hearts shall be staunched, and their broken spirits healed by His loving mercy. Far back in their minds will be a sweet and secret feeling which they cannot explain to themselves, but which exercises a calm influence in the most dismal circumstances of life.

The value of the world sinks, all those things which appeared indispensable to their happiness become worthless, and the mind, without an effort, turns to its only Comforter, and rests in the contemplation of God. It was to arrive at such a state as this that St. Paul bids us set our affections upon things above, and not on things of the earth. It was for such happiness that Christ died, imploring us to strive after a spiritual union with Himself and His Father.

But to understand sorrow properly we must look at Christ in the agony of His last hours, in the Garden of Gethsemane. Then shall we see how lonely sorrow is. Not one friend of all those whom He had taught and benefited was there to help Him. The heart truly knoweth its own bitterness, and a stranger doth not intermeddle with its grief. Thoughts are in our hearts in our time of deepest woe which we can tell to no one, and which those nearest and dearest to us would never guess.

> Not even the tenderest heart, and next our own,
> Knows half the reasons why we smile or sigh.

Thoughts possibly of anger against the God who has given us this bitter suffering; of restless discontent with Him and all the world are surging in our hearts. Such a time as this is the turning point of the spiritual life, and on how it is borne depends our future in this world and the next. Shall we be hardened or softened? Pray God that we may not be driven into farther disobedience and revolt. Blessed is the man who finds that in the loneliness of his sorrow he is alone with God too. Then he will pray with all his heart, he will cease to fear, and will lean on that Divine help.

